# The Effect of Neutral Recombination Variation on Genome Scans for Selection

**DOI:** 10.1534/g3.119.400088

**Published:** 2019-04-10

**Authors:** Katie E. Lotterhos

**Affiliations:** Department of Marine and Environmental Sciences, Northeastern University, 430 Nahant Road, Nahant, MA 01908

**Keywords:** principal components, adaptation, population structure, linkage disequilibrium

## Abstract

Recently, there has been an increasing interest in identifying the role that regions of low recombination or inversion play in adaptation of species to local environments. Many examples of groups of adapted genes located within inversions are arising in the literature, in part inspired by theory that predicts the evolution of these so-called “supergenes.” We still, however, have a poor understanding of how genomic heterogeneity, such as varying rates of recombination, may confound signals of selection. Here, I evaluate the effect of neutral inversions and recombination variation on genome scans for selection, including tests for selective sweeps, differentiation outlier tests, and association tests. There is considerable variation among methods in their performance, with some methods being unaffected and some showing elevated false positive signals within a neutral inversion or region of low recombination. In some cases the false positive signal can be dampened or removed, if it is possible to use a quasi-independent set of SNPs to parameterize the model before performing the test. These results will be helpful to those seeking to understand the importance of regions of low recombination in adaptation.

A recent focus of evolutionary biology has been to understand the genetic basis of adaptation. Recent studies have uncovered a range of complex genetic architectures underlying traits, and the idea that adapted loci may be co-located and physically linked in the genome has received a large amount of attention. These observations range from extended signals of selection deemed “genomic islands” (Feder *et al.* 2012), to clusters of tightly linked loci located within inversions deemed “supergenes” (Schwander *et al.* 2014; Thompson and Jiggins 2014), to an important role of restricted recombination in local adaptation within species and maintaining boundaries among species (Noor and Bennett 2010; Berner and Roesti 2017). These empirical observations are expected based on theory. Theory predicts that rearrangements can bring locally adapted alleles together (Yeaman 2013) and that linked alleles have increased establishment probability (Yeaman *et al.* 2016). Theory also predicts that rearrangements that capture locally adapted alleles will prevent shuffling of alleles in heterozygotes, and that regions of suppressed recombination results in reduced gene flow that enables locally adapted alleles to evolve (Kirkpatrick and Barton 2006; Guerrero *et al.* 2012; Fuller *et al.* 2018).

Many of these observations of extended signals in the genome are based on the statistical analysis of population-genetic datasets using genome scans. ***Genome scans*** are statistical tests used to infer the genetic loci either affected by selection or that affect a trait (Table 1). Genome scans can be divided into three groups: those that seek to identify loci more differentiated among populations that expected from neutrality (differentiation outlier methods, Hoban *et al.* 2016), those that seek to identify loci that are associated with a variable such as a phenotype or the environment where the individual was sampled (association methods, Bush and Moore 2012; Korte and Farlow 2013; Rellstab *et al.* 2015), and those that seek to characterize allele frequency or haplotype shifts due to positive selection acting within the sample (sweep methods, Pritchard and Di Rienzo 2010; Pritchard *et al.* 2010; Schlamp *et al.* 2016). Despite the theoretical expectations that we should find signals of selection within inversions or regions of low recombination, we still have a poor understanding of how genomic heterogeneity, such as variation in recombination rate, may produce false positive signals in the very regions we expect to find adaptive signals. Every genome scan method outputs a ***test statistic*** (Table 1) for each locus, or a numerical summary of the signal at that locus, and the statistic itself or the significance of that statistic can be biased if the data violates assumptions.

**Table 1:** Explanation of terms used in this study

Term	Description
Genome scan	A statistical test used to infer the genetic loci either affected by selection or that affect a trait
Test statistic	The numerical summary calculated for each locus that reduces the data to one value that can be used to perform the hypothesis test. The relationship between the test statistic and the *P*-value for that test statistic depends on the shape of the distribution used to model the null hypothesis (*e.g.*, neutral parameterization).
Quantitative trait nucleotide (QTN)	A causal allele that has an additive effect on a quantitative trait
Linkage disequilibrium (LD)	Non-random association of alleles at different loci
Neutral parameterization	The process of calibrating or calculating the distribution of the test statistic expected under neutrality (*i.e.*, the neutral demographic process)
Neutral parameterization with all SNPs and genome scan on all SNPs (“naive”)	When based on all SNPs, neutral parameterization may be biased due to non-independence among SNPs due to linkage disequilibrium. Since this is the default for many programs, it is referred to as the “naive” approach. But note that for some genome scans, this is the only option.
LD thinning, SNP thinning, thinned SNPs	Move along a genome in a sliding window and reduce the set of SNPs to those that have reduced linkage disequilibrium with each other
Neutral parameterization with thinned SNPs and genome scan on thinned SNPs	When based on a set of SNPs thinned for LD, neutral parameterization is less likely to be biased due to linkage disequilibrium among SNPs. However, trimming removes many causal SNPs, and can greatly reduce the probability of finding causal SNPs in the genome scan. For this reason, this approach was not evaluated.
Neutral parameterization with thinned SNPs and genome scan on all SNPs (“best practice”)	Neutral parameterization on a set of thinned SNPs is less likely to be biased due to linkage disequilibrium among SNPs. This parameterization can then be used when performing the genome scan on the entire set of SNPs.

Unfortunately, the rate at which new genome scan tests are being developed is rapidly eclipsing our understanding of their performance, and the effects of heterogeneity in the data (such as recombination variation) have not been well evaluated with simulations. Linkage disequilibrium (LD) is the non-random association of alleles at different loci (Robbins 1918). Regions of extensive LD may be found following hard selective sweeps as a new beneficial mutation arises in frequency (Maynard-Smith and Haigh 1974; Charlesworth *et al.* 1997) as well as around regions of low recombination such as centromeres or inversions (Kirkpatrick and Barton 2006; Berner and Roesti 2017). In this case, the potential issue is that the extensive LD that is expected to arise around a nucleotide due to strong selection is the same extensive LD that may be observed in neutral regions of low recombination, and that test statistics based on the allele frequency spectrum or haplotype structure (Messer and Petrov 2013; Schlamp *et al.* 2016) may be similar between these two types of regions. Often measures of nucleotide diversity and measures of the allele frequency spectrum are correlated with recombination variation in genomes, but this is confounded with the action of positive selection and background selection (Charlesworth *et al.* 1993; Nachman 2001; Charlesworth 2012). Whether tests for selective sweeps have false positive signals in neutral regions of low recombination has not been systematically evaluated with simulations.

A second potential issue is raised by a handful of empirical studies that have found that even when data across the genome is used, long-range LD at a particular locus can bias estimates of population structure and neutral demography (Price *et al.* 2006; Corbett-Detig and Hartl 2012; Abdellaoui *et al.* 2013; Privé *et al.* 2018). Accurately correcting for neutral demography in genome scans is important because non-independence among populations can create false positive signals. For example, early differentiation outlier methods basically assumed that migration was equal among all populations (*e.g.*, all populations were equally related to each other, Lewontin and Krakauer 1973; Beaumont and Nichols 1996). These methods had high false positive rates because they did not account for complex demographic histories that create varying degrees of relatedness among populations in the data (Bonhomme *et al.* 2010; De Mita *et al.* 2013; Lotterhos and Whitlock 2014, 2015; Hoban *et al.* 2016). It was also realized that association methods that did not correct for demographic history would exhibit false positive signals because of spatial autocorrelation in allele frequencies (Meirmans 2012). For this reason, most of the recently developed differentiation outlier and association methods use ***neutral parameterization*** (Table 1, *sensu* Lotterhos and Whitlock 2014) to calibrate or calculate the distribution of the test statistic expected under the neutral demographic process. Because genome scans must correct for population structure in the statistical test, long-range LD that distorts estimates of population structure will also distort the significance of the test statistic (Price *et al.* 2008). This suggests that distortion in population structure due to LD may also bias the results of differentiation outlier and association tests that require neutral parameterization, but this also has not been systematically evaluated with simulations.

Overall, the effects of genomic heterogeneity in recombination and long-range LD on neutral parameterization, as well as on estimates of population structure, have not been well characterized. This is in part because most of the evaluative studies have simulated unlinked, independent loci and are not useful for evaluating this problem (e.g., Price *et al.* 2006; Novembre and Stephens 2008; McVean 2009; De Mita *et al.* 2013; Lotterhos and Whitlock 2014, 2015; Forester *et al.* 2016, 2018; Martins *et al.* 2016; Luu *et al.* 2017). Here, I develop a novel set of simulations to evaluate how genomic heterogeneity in recombination affects the ability of genome scans to identify partial sweeps from a new mutation (Hudson *et al.* 1994; Voight *et al.* 2006; Pritchard *et al.* 2010), complete sweeps from a new mutation (Smith and Haigh 1974), or causal quantitative trait nucleotides (***QTNs***, Table 1) that affect a trait under spatially heterogeneous selection. One novel aspect of the simulations is that each of the 9 linkage groups (LGs) simulated were subject to a kind of realism expected in actual genomic data (*e.g.*, neutrality, QTNs, selective sweeps, an inversion, a centromeric region, and recombination variation). Having genomic heterogeneity in the same simulation is essential not only for simulating realistic genealogies (as determined by the contributions to fitness from different regions of the genome), but it is also essential for the evaluation of genome scans because false positive signals or biased neutral parameterization will affect the false discovery rate. As shown below, the genomic heterogeneity does confuse some analyses, but other analyses are unaffected. It is precisely this case when genomic heterogeneity confuses analysis that demonstrates how caution must be applied when analyzing genomic data.

I use a basic demography that results in an isolation-by-distance pattern in the data to explore the effects of genomic heterogeneity, rather than the effects of different population demographies. While the genomic heterogeneity in LD among SNPs in the simulations is caused by variation in recombination rates among proximate SNPs, similar heterogeneity might be present in a dataset which contained a mixture of random sets of SNPs from the entire genome and long sequences of proximate SNPs obtained by sequence capture.

## Methods

### Simulations

**Figure 1:**
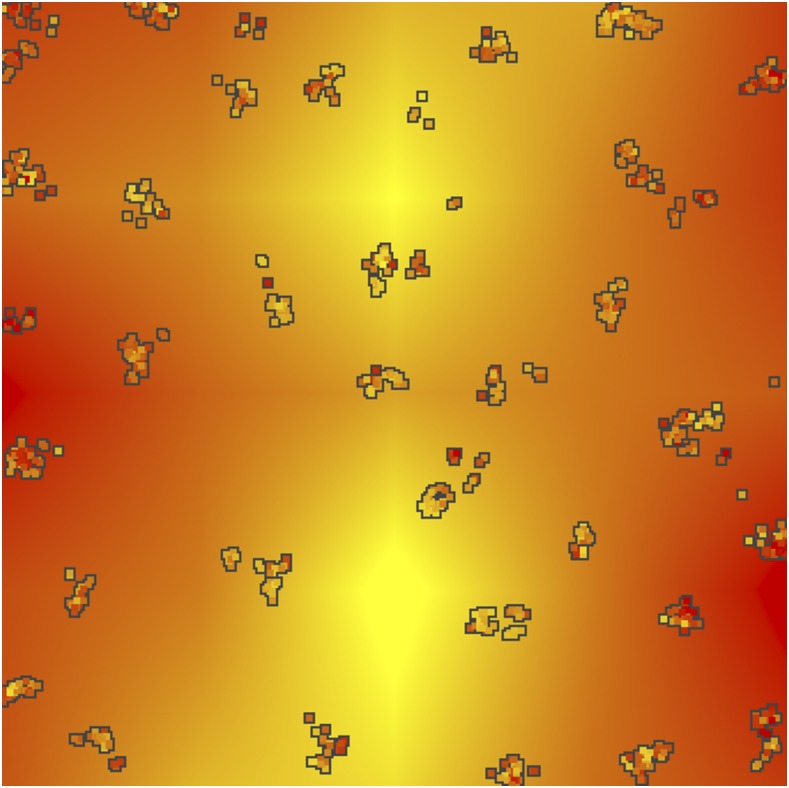
Example landscape simulation. Each box is an individual, colored by their phenotypic value. The background is the selective environment. This output was generated after 1900 generations of selection by the environment, resulting in a correlation of 0.52 between the phenotype and the environment.

I conducted 200 replicate forward-time simulations of a metapopulation adapting to a heterogeneous spatial environment (Figure 1) with SLiM v. 3.2 (Haller and Messer 2017) to create SNP data for each individual. The simulations resulted in a population that had isolation-by-distance structure along an environmental gradient (*e.g.*, isolation by environment, Wang and Bradburd 2014). For simplicity in interpreting the results, only one type of genomic heterogeneity was simulated on each LG, such that each LG evolved approximately independently. Each of the 9 LGs were 50,000 bases and 50 cM in length. The base recombination rate *N_e_r* = 0.01 (unless manipulated as described below) gave a resolution of 0.001 cM between proximate bases. The recombination rate was scaled to mimic the case where SNPs were collected across a larger genetic map than what was simulated (similar to a SNP chip), but still low enough to allow signatures of selection to arise in neutral loci linked to selected loci (in the simulations 50,000 bases / (*r* = 1e-05) * 100 = 50 cM; in humans 50,000 bp would correspond to 0.05 cM). Thus, SNPs at the opposite ends of linkage groups were likely to have a recombination rate between them of 0.5 (unlinked), but there would otherwise be some degree of linkage among SNPs within linkage groups. For all LGs, the population-scaled mutation rate *N_e_*μ equaled 0.001. For computational efficiency, 1000 individuals were simulated with scaling of mutation rate and recombination rate as described above (Fisher 1930; Wright 1931, 1938; Crow and Kimura 1970; Bürger 2000). In the first generation, individuals were placed randomly on a spatial map between the coordinates 0 and 1. Individuals dispersed a distance given by a bivariate normal distribution with zero mean and variance $\sigma_{d}$ (Table 2).

**Table 2:** Parameters used in the simulations and their values

Symbol	Value	Description
*N_e_*	1000	Population size
σ_d_	0.004	Standard deviation of dispersal
σ_S_	3.0	Strength of stabilizing selection for first 4*N_e_* generations
σ_K_	0.05	Strength of stabilizing selection after 4*N_e_* generations
σ_C_	0.07	Standard deviation of competition function for total interaction strength
σ_M_	0.5	Standard deviation of mating function for total interaction strength
μ	10^−6^	Mutation rate
*r*	10^−5^	Base recombination rate
σ***_QTN_***	0.7	Standard deviation of QTN effect sizes

A unique aspect of these simulations is that individuals experienced six components of fitness to reflect a core set of biologically realistic pressures acting on the genome: (i) local adaptation of QTNs with additive effects on a phenotype subject to selection by a heterogeneous environment, (ii) competition, (iii) mating success, (iv) a beneficial new mutation (hard sweep) at a single site introduced 300 generations before sampling, which was enough time for the sweep to be near or at fixation for varying lengths of time (“full sweep”), (v) a beneficial new mutation (hard sweep) at another site introduced 60 generations before sampling, which was enough time for the sweep to typically reach a frequency from 0.5-0.85 (“partial sweep”), and (vi) weak negative frequency dependent selection on the inversion, which was included to maintain the inversion as polymorphic in the population (but was unrelated to local adaptation to the environment). Total fitness was calculated as the multiple of the fitness components after they were scaled to relative fitness for that component. The parameters chosen for competition and mating (described in Appendix) resulted in local neighborhoods or clusters of individuals (Figure 1), which could later be used to assign individuals to populations for methods that required it. The remaining fitness components are described below in the “Genetic Map” section.

I tested for coalescence with preliminary simulations and found for some replicates that even 50N generations of burn-in was not enough time for the metapopulation to coalesce. Therefore, I only simulated non-neutral mutations in SLiM with tree sequencing implemented to record the geneology, and then used recaptitation with pyslim (v0.1) and msprime (v0.6.1) to reconstruct the ancestry of the initial genomes using the coalescent and to add neutral mutations (Haller *et al.* 2018; Kelleher *et al.* 2018). Note that at this time recapitation of a SLiM simulation can only be performed on a uniform recombination map. To evolve more realistic patterns of haplotype diversity under recombination variation, the SLiM simulation was run without spatially heterogeneous selection for 10*N* generations, followed by a period of 2*N* generations during which the metapopulation experienced spatially heterogeneous selection by the environment. After recaptitation and the addition of neutral mutations, a vcf file was produced for subsequent analyses with genome scans. See *Data Availability* for scripts and code.

### Data filtering

VCF files produced from the simulations were first filtered for individuals that were related and then for loci that had MAF < 0.01 with the R package vcfR (Knaus and Grunwald 2016). Relatedness was calculated using the statistic of Lynch and Ritland (Lynch and Ritland 1999) with the R package related for a subset of 800 SNPs with MAF > 0.05, and for pairs of individuals with a pairwise relationship coefficient greater than 0.5, one individual from the pair was removed. The test statistics described below were then calculated from this filtered set of data.

### Genetic map and fitness components

#### LG-1 and LG-2: Neutral:

**Figure 2:**
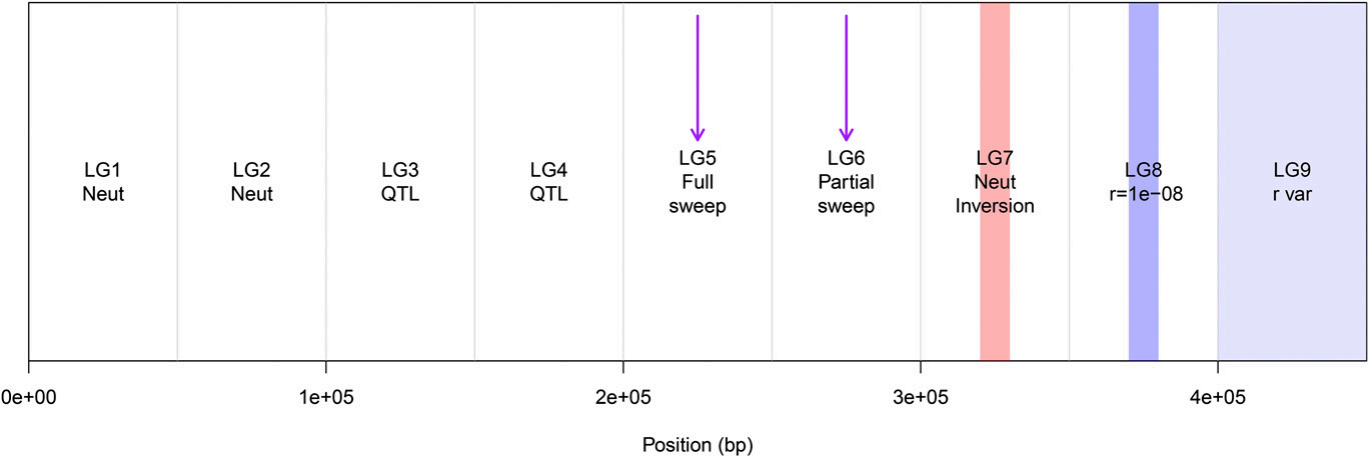
Example genetic map with 9 linkage groups (LGs), each 50 cM long. LG-1 and LG-2 were neutral; on LG-3 and LG-4 quantitative trait loci (QTL) were allowed to evolve (but exact locations and effect sizes depended on the simulation); on LG-5 and LG-6 a hard selective sweep from a new mutation occurred at the purple arrow; on LG-7 a neutral inversion was simulated in the center; on LG-8 a region of low recombination (3 orders of magnitude lower than the base rate) was simulated in the center; and on LG-9 recombination variation was simulated randomly.

The first two linkage groups were simulated under neutrality as described above (Figure 2). No loci in these regions affected fitness components.

#### LG-3 and LG-4: Quantitative trait nucleotides:

On these linkage groups QTNs that had additive effects on the trait could evolve with the probability of a new mutation being a QTN equal to 0.02 (Figure 2). If a new mutation was a QTN, the effect size was drawn from a distribution with mean 0 and variance σ*_QTN_*. In this manner genetic architecture (the distribution of effect sizes and linkage relationships among causal mutations) was allowed to evolve. Because they were subject to spatially heterogeneous selection, QTNs that evolved to explain a major proportion of the genetic variance created signatures that could be detectable by differentiation outlier methods and genetic-environment associations (GEAs) (Hoban *et al.* 2016). Because they had additive effects on phenotypes, they could also be detectable by genome-wide association studies with phenotypes (GWAS) (Bush and Moore 2012).

To simulate local adaptation, the trait was subject to spatially heterogeneous stabilizing selection with the optimum for each location in space dependent on the environment (Figure 1). To allow some standing genetic variation at the QTNs to evolve prior to the onset of heterogeneous selection, the phenotype was under weak stabilizing selection with a mean trait optimum *θ =* 0 and variance $\sigma_{S}$ for the first 10*N* generations. At 10*N* generations selection by the environment was initiated. For each location, the environmental optimum was randomly generated at 25 locations on the grid by adding random variation (σ = 0.5) to a longitudinal cline (from -1 in the west, to 1 in the center, and -1 in the east) to mimic a mountain range, and then interpolated to determine the environmental optimum *θ_xy_* for the trait at any *{x,y}* location (Figure 1). When selection to the environment was initiated, the environmental optimum at each location changed from the historical optimum to the new optimum linearly for 100 generations, and then remained at the new optimum for the remainder of the simulation.

For each individual in each generation, the local-adaptation fitness-component was determined by a Gaussian function given the difference between the individual’s phenotype and the optimum at that location, with the strength of stabilizing selection as the variance of the Gaussian function $\sigma_{K}$. The fitness component for individual *i* at location *{x,y}* was:

$$\omega_{QTN_{i}}=1.0+\frac{1}{\sqrt{2\pi \sigma_{K}^{2}}}e^{\frac{{\left(z_{i}-\theta xy\right)}^{2}}{2\sigma_{K}^{2}}}$$ (1)

where *z_i_* is the phenotype (calculated from the sum of QTN effect sizes) of individual *i*, and *θ_xy_* is the optimum at the location where the individual was located.

#### LG-5 and LG-6: Selective sweeps:

In the center of LG-5 and LG-6, positive selection on a single new mutation was simulated to create the signature of a hard selective sweep (Figure 2). Because these sites arose in frequency across the metapopulation, it was not going to be detectable by differentiation outlier, GEA, or GWAS methods. Instead, these sites would be detectable by methods based on the allele frequency spectrum or haplotype-based methods (Messer and Petrov 2013; Schlamp *et al.* 2016). I simulated two strongly selected mutations with the objective of creating obvious signatures that should be detectable by methods based on allele frequency spectrum or linkage disequilibrium. Each positively selected mutation was dominant and had a selection coefficient *s_sweep_* = 0.5. Since some genome scans are sensitive to whether or not the sweep fixed, I simulated an older sweep on LG-5 that was at or near fixation by the end of the simulation (“full sweep”, introduced 300 generations before the end of the simulation) and a more recent sweep on LG-6 that typically rose to a frequency of 0.5-0.85 (“partial sweep”, introduced 60 generations before the end of the simulation). To ensure that the sweep was present at the end of the simulation, it was reintroduced if it was lost to drift within 20 generations of first introduction.

#### LG-7 Neutral with a large inversion:

A 10000 base pair region in the center LG-7 was simulated as a large inversion. While in nature recombination may occur between inverted and ancestral haplotypes in a heterozygote, a single crossover would result in inviable gametes that lack critical genes. Since this process effectively results in essentially no recombination between inversion and ancestral haplotypes, for computational simplicity recombination was allowed between homozygotes but completely suppressed between heterozygotes.

To ensure that the inversion was segregating in the population at the end of the simulation, the inversion was maintained under weak negative frequency dependent selection. Individuals without the inversion had a relative fitness equal to 1.0, while individuals with the inversion had a fitness component that depended on the frequency of the inversion in the population *f*: $\omega_{inversion_{}}$ = 1.0 - (*f* - 0.5) * 0.1. Thus, at any point in time the fitness component of the rarer haplotype was slightly higher than the common haplotype. Note however that the fitness of the inversion was not related to local adaptation to the environment, and all mutations within the inversion were neutral. These parameters resulted in a range of minor allele frequencies of the inversion haplotypes across replicate simulations (see Results).

#### LG-8 Neutral with a large region of low recombination:

On, LG-8 a stretch of 10,000 bases at the center of the LG was simulated with a recombination rate of 10^−8^, which was three orders of magnitude lower than the base recombination rate, but would have been higher than recombination between the two inversion haplotypes where recombination was completely suppressed.

#### LG-9 Neutral with recombination variation:

LG-9 was simulated with random recombination rate variation, where 9 breakpoints were randomly chosen from a uniform distribution and the recombination rates for the 10 resulting regions were generated by 10^-^*^b^*, where *b* was a random normal variable with mean 5 and standard deviation of 2. This generated recombination rates between ∼0.01 and 10^−9^ at random breakpoints, with a mean of the base recombination rate at 10^−5^.

### Population structure and neutral parameterization

Because of the potentially confounding effects of long-range LD, many population-structure methods recommend that an “independent” set of SNPs be used to estimate population structure (Bradburd *et al.* 2018). Here I will use the term “quasi-independent,” because all SNPs are located within the same genome and can never be truly independent. A common way of obtaining a quasi-independent set of SNPs is to thin for linkage disequilibrium (***LD or SNP thinning***, Table 1), which typically moves along a genome in a sliding window and thins SNPs based on linkage disequilibrium with each other. This may be based on a combination of (i) “pruning,” which sequentially scans the genome and performs pairwise thinning based on a given threshold of correlation, (ii) “clumping,” which may incorporate some information about the importance of SNPs based on summary statistics, and (iii) removing SNPs in long-range LD regions (Privé *et al.* 2018). While SNP thinning is a recommended practice for obtaining a set of SNPs to estimate population structure, it is unclear what the best practices are for genome scans because SNP thinning will (i) remove the characteristic signature that selection will leave in the genome and (ii) potentially remove causal or adaptive SNPs (Table 1: “***Neutral parameterization on thinned SNPs and genome scan on thinned SNPs***”). Methods vary widely in how they control for structure, their flexibility, and their guidelines for users. For example, some methods allow the user to use one set of SNPs for neutral parameterization and perform the statistical test on another (more complete) set of SNPs, and clearly recommend that a random and independent set be used for the first step (*e.g.*, Günther and Coop 2013) (Table 1, “***Neutral parameterization with thinned SNPs and genome scan on all SNPs***”, “best practice”). Other methods could be implemented in these two steps - but that is not the default and best practices are not well articulated in the resources for users - and so naïve users tend to use the entire set of SNPs for neutral parameterization and to perform the test (Table 1, “***Neutral parameterization on all SNPs and genome scan on all SNPs***”, “naïve approach”). Note that for some genome scan approaches like haplotype-based statistics, this latter approach is the only option. In many cases, the best practices for implementing genome scans in the presence of long-range LD are unclear.

When applicable, the “naïve approach” for the method was run on all SNPs with MAF > 0.01. When applicable (as noted for each method below), the “best practice” involved implementing the statistical tests in two steps (Table 1). In step 1, the whole genome data were thinned for LD to obtain a quasi-independent set of SNPs, and this set of *thinned* SNPs were used for neutral parameterization. In step 2, these null parameters were used to calculate the test statistic for *all* SNPs. SNP thinning was implemented in R packages bigsnpr and bigstatsr with the function snp_autoSVD, which uses sliding windows to remove SNPs correlated greater than 0.2 with the SNP with the highest MAF in that window and removes regions with putative long-range LD (Privé *et al.* 2018).

Because the issues with some genome scans arise through how they capture population structure via the process of neutral parameterization, I also evaluate the “naïve approach” (all SNPs with MAF > 0.01) and the “best practice” (thinned SNPs, Table 1) on population structure estimates from principal components. Principal components analysis was conducted on the SNP matrix (coded as 0, 1, or 2) using the function pcadapt() in the R package pcadapt v3.0.4 (Luu *et al.* 2017). This function output the score for each diploid individual (`$scores`) and the loading of individual SNP (`$loadings`) onto each PC axis. I evaluated the degree to which scores reflected the isolation-by-distance population structure that evolved on the landscape, as well as the degree to which loadings were sensitive to genomic variation in recombination rate.

### Genome scan approaches

I compared different methods for detecting selection from genomic data, including differentiation outlier tests (Hoban *et al.* 2016), genome-wide association studies with phenotypes (Bush and Moore 2012; Korte and Farlow 2013), genetic-environment associations (Rellstab *et al.* 2015), and statistics for detecting selective sweeps based on shifts in haplotype frequencies (Pritchard and Di Rienzo 2010; Schlamp *et al.* 2016). When applicable, a method was run with a “naïve approach” and the “best practice.” The “best practice” was determined by any of the following: (i) following recommendations in the citation and the user guides, (ii) communication with the developer, and (iii) preliminary data analysis on a subset of simulations.

#### Selective sweep methods:

The statistics evaluated are designed to detect the shifts in haplotype frequencies around the new mutation under positive selection (LG-5 and LG-6 in the simulations). I evaluated three statistics:

- iHS: integrated haplotype score; a measure of the amount of extended haplotype homozygosity (Voight *et al.* 2006);
- *H_12_*: estimates haplotype homozygosity by combining the frequencies of two most frequent haplotypes into a single frequency and adding it to the total haplotype homozygosity (Messer and Petrov 2013; Garud *et al.* 2015); and
- *H_2_/H_1_*: compares the haplotype homozygosity using all but the most frequent haplotype to the total haplotype homozygosity (Messer and Petrov 2013; Garud *et al.* 2015). This value is expected to be smaller for hard sweeps, so was transformed to -log_10_(*H_2_/H_1_*) for performance evaluation (see Supplementary Materials).

*iHS* is known to have lower power than *H_12_* when the sweep is near fixation (Tang *et al.* 2007; Schlamp *et al.* 2016). Also, *iHS* is standardized to correct for variation in recombination rate over a range of SNPs with similar derived allele frequencies (Voight *et al.* 2006), but *H_12_* and *H_2_/H_1_* are not. Statistics were trimmed within 1000 bases of the ends of the linkage groups to reduce potential biases there. Because these methods are based on haplotypes, they could only be implemented on the complete set of SNPs. For details of all calculations see the Supplementary Materials.

#### Differentiation outlier methods:

The differentiation outlier methods are designed to detect loci that are subject to spatially heterogeneous selection and evolve to be differentiated above and beyond that expected by neutral demographic history (QTNs on LG-3 and LG-4). I compared three methods that differ in the way that they correct for population structure:

- OutFLANK: identifies *F_ST_* outliers after estimating the neutral parameters on the *F_ST_* distribution (Whitlock and Lotterhos 2015);
- PCAdapt: identifies outliers along the principal components that describe structure (Duforet-Frebourg *et al.* 2014; Luu *et al.* 2017); and
- *X^T^X*: identifies outliers along the covariance in allele frequencies among populations based on the model of Bayenv2 (Günther and Coop 2013) as implemented by Gautier (2015).

For each statistic, the “naïve approach” was evaluated using the results from running the algorithm on all SNPs. The “best practice” was evaluated using the results from running the algorithm in two steps: first, using a quasi-independent set of thinned SNPs for neutral parameterization, and then second, using that parameterization to run the model and obtain *P*-values or test statistics for all SNPs. See Supplemental Materials for details on all calculations.

#### Genome-wide association (GWAS) methods:

The GWAS methods are designed to detect loci that have effects on phenotypes and evolve to be associated with the phenotypes (QTNs on LG-3 and LG-4). I compared two methods that adjust for coefficient inflation in a latent factor mixed model (LFMM) association between genotypes and phenotypes: ridge regression and lasso (Caye *et al.* 2019; François and Caye 2018). LFMMs model unexplained variation with latent factors, which are estimated jointly with the main effects in the model. Thus, neutral parameterization with these methods happens internally (*e.g.*, the latent factors and model coefficients are estimated jointly), and so the algorithms can only be run in one step on the set of all SNPs. To accurately estimate latent factors that capture the genetic population structure in a GWAS, genotype is modeled as a function of phenotype (François and Caye 2018). I tested two different methods for adjusting for coefficient inflation in large data sets with collinear predictor variables (Caye *et al.* 2019):

- ridge regression, which adjusts all model coefficients by a shrinkage term; and
- lasso, which adjusts for coefficient inflation by minimizing the residual sum of squares with a penalty.

More details can be found in the Supplementary Materials.

#### Genetic-environment association (GEA) methods:

The GEA methods are designed to detect loci that are subject to spatially heterogeneous selection by a specific environment and evolve to be associated with that environment (QTNs that evolve on LG-3 and LG-4). I compare four methods that measure the association between the allele frequency and an environmental variable:

- latent factor mixed Bayesian hierarchical model: measures environment-allele association corrected for structure with latent factors (Frichot and François 2015);
- Bayes Factor from BayPass: measures strength of evidence of an association, corrected for structure with population covariance matrix (Gautier 2015). The “naïve approach” and “best practice” were implemented as described for *X^T^X* above;
- Spearman’s ρ: uncorrected association between allele frequency and the environment; and
- redundancy analysis: a method to extract and summarize the variation in a set of response variables (SNPs) that can be explained by a set of explanatory variables (environmental variables) (Legendre and Legendre 2012). Performance was evaluated using the loading of SNP on the constrained axis (*e.g.*, the environmental predictor) following Forester *et al.* (2018); note this approach does not correct for structure.

For details on these calculations see Supplementary Materials.

### Comparison of performance

Causal SNPs were counted as true positives if they contributed more than 1% to the additive genetic variance of the trait. Because SNPs were filtered for final data analysis using standard cutoffs (MAF > 0.01), some causal SNPs were below this threshold (rare alleles). Some of these rare alleles were of small effect or just recently introduced in the population, thus contributing very little to the overall genetic variance of the trait. The proportion of additive genetic variance for causal SNPs was approximated as the additive genetic variance for SNP *i* standardized by the total additive genetic variance:

$$\frac{\alpha_{i}^{2}p_{i}\left(1-p_{i}\right)}{\sum_{i=i}^{N}\alpha_{i}^{2}p_{i}\left(1-p_{i}\right)},$$

Where *α_i_* is the effect size and *p_i_* is the allele frequency of the derived allele at SNP *i*. Causal SNPs whose contribution to the additive genetic variance was greater than a proportion of 0.01 were then counted as true positives (hereafter: “causal SNPs”). Note that some of these causal SNPs were rare alleles (*e.g.*, MAF < 0.01) of large effect, and so they were not present in the set of analyzed SNPs but they were still counted in the calculation of error rates because they explained more than 1% of the additive genetic variance (see Results).

The performance of each metric was then summarized as the area under the curve of the precision-recall graph (AUC-PR) (Davis and Goadrich 2006). This is preferred to the area under the curve of the Receiver Operating Characteristics graph (AUC-ROC) graph (Fawcett 2004), because in biological applications when the number of samples in each classifier is imbalanced (for example in these simulations there are many neutral loci and only a few selected loci), a method can achieve a high AUC-ROC even when the majority of positive hits are false positives (Saito and Rehmsmeier 2015). In these cases AUC-PR more accurately captures performance. An AUC-PR = 1 represents the case when all causal SNPs have higher scores than neutral SNPs. The AUC-PR that is expected by random is equal to the proportion of causal loci in each dataset, which varied slightly from simulation to simulation but was generally less than 1%. The AUC-PR was calculated with the R package PRROC (Grau *et al.* 2015) using the continuous interpolation method (Boyd *et al.* 2013; Keilwagen *et al.* 2014).

I summarized performance of each method for the ability to detect (i) all causal QTNs and (ii) a 2000 bp region on either side of a selective sweep (note in this latter case the sweep nucleotide was not always in the dataset because it was at or near fixation). The distribution of AUC-PR over replicate simulations was visually compared among methods with boxplots. I inspected how signals in different regions of the genome affected the performance of each metric by calculating the empirical cumulative distribution function for neutral loci (ecdf() function in R) and evaluating the average quantile of different regions of the genome (*e.g.*, sweeps, QTNs, inversion, etc.) relative to this empirical neutral distribution (Lotterhos and Whitlock 2014).

### Data Availability

To facilitate validation of new methods and their sensitivity to genomic realism, I have created a github group called “Test the Tests.” Within this group is a repository for this study, https://github.com/TestTheTests/TTT_RecombinationGenomeScans, that includes all the scripts needed to recreate the simulations and figures presented in this manuscript. The repository is structured so that the simulations may be easily analyzed by new methods, and that the results may be easily added and visually compared using the same metrics presented here. In addition the repository and “.trees” files from the simulations are archived on Dryad at https://doi.org/10.5061/dryad.rj0kj10 (Lotterhos 2019). Supplemental material available at FigShare: https://doi.org/10.25387/g3.7973438.

## Results

After filtering for minor alleles, on average datasets had 6647 SNPs. On average, 5% of SNPs were located within the non-adaptive inversion on LG-7 and 2% in the region of low recombination on LG-8. The simulation parameters resulted in a range of minor haplotype frequencies in the inversion and a range of ages for the origin of the inversion (Supplemental Figure S1).

On average 9.6 causal SNPs evolved that contributed more than 1% to additive genetic variance (V_A_) in the trait. The distribution of causal allele frequencies and their effect sizes are shown in Supplemental Figure S2. Typically, 4-13 SNPs explained 80–98% of V_A_ (based on 0.05 and 0.95 quantiles), and the locus in each simulation that explained the most genetic variance explained on average 45%. Rare alleles of large effect (that were filtered for analysis because they were rare) typically left 3% of V_A_ unexplained, but this could be as high as 15–20% in a few simulations (Supplemental Figure S3).

The full hard selective sweep generally was at or near fixation (frequency > 0.9) by the end of the simulation, and the mutations that fixed were fixed for 1-200 generations (up to 0.2N generations, Supplementary Figure S4 A, B). The partial hard sweep generally reached an allele frequency of 0.5-0.85 by the end of the simulation but never fixed (Supplementary Figure S4 C).

### Effect of inversions and thinning on population structure

#### PC scores of individuals on PC axes:

**Figure 3:**
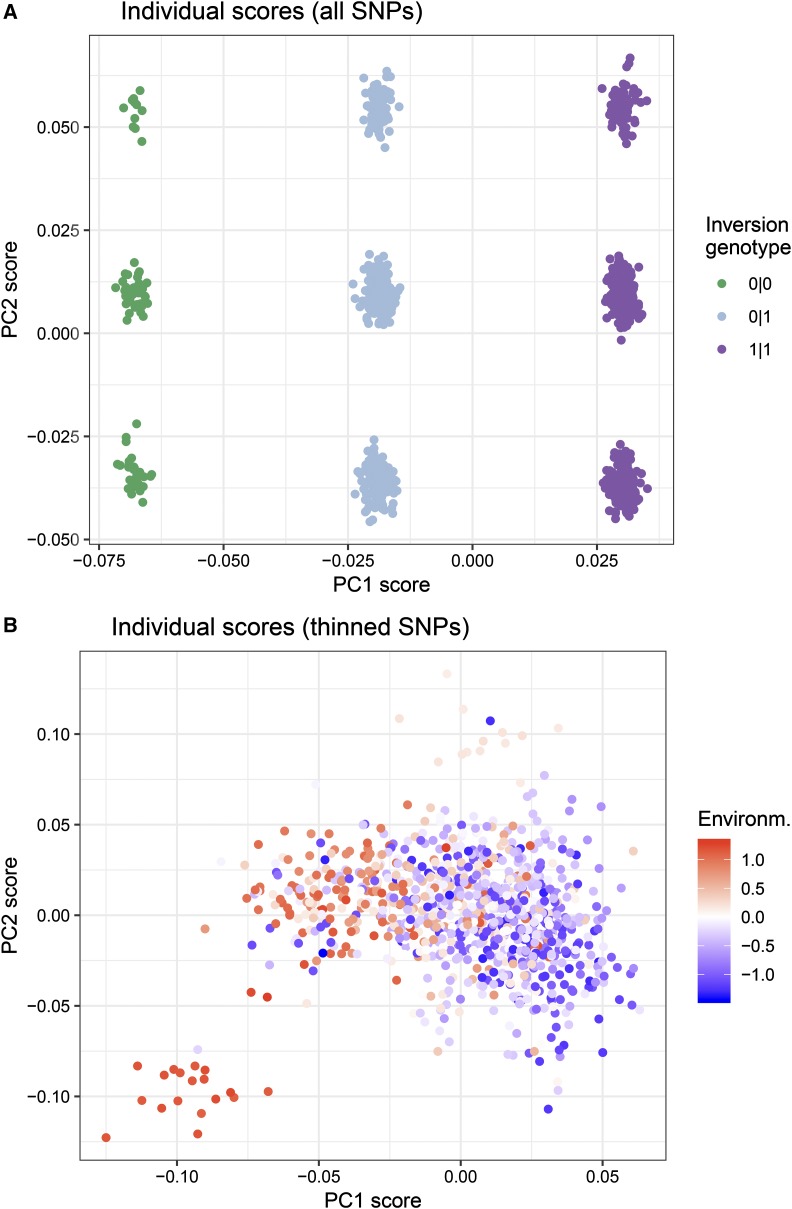
PC scores of individuals on PC axes for one replicate simulation. A) PC scores calculated from all loci. Structure along PC1 was determined by the number of copies of the inversion (“0|0” lacked inversion, “0|1” inversion heterozygote, and “1|1” inversion homozygote). In this simulation, structure along PC2 was determined by haplotype in the region of low recombination on LG-8. B) PC scores calculated from a quasi-independent set of thinned SNPs, which resulted in PC1 reflecting the isolation-by-environment structure in the data.

When all the SNPs were used to conduct a PCA, scores of individuals along PC1 depended on their inversion haplotype on LG7 in the majority of replicate simulations (Figure 3A). This occurred despite the SNPs in this region representing on average 5% of the total number of SNPs in the data. Their scores along PC2 depended on the simulation, but generally were determined by their haplotype in the region of low recombination on LG8 or in the region of recombination variation on LG9. This means that when all SNPs in the data were used, the population structure was reflecting these regions in the genome rather than the isolation-by-environment pattern in the data.

In contrast, when a set of quasi-independent thinned SNPs were used, the scores of individuals along PC1 reflected the isolation-by-environment pattern in the data (Figure 3B).

#### PC loadings of loci onto PC axes:

**Figure 4:**
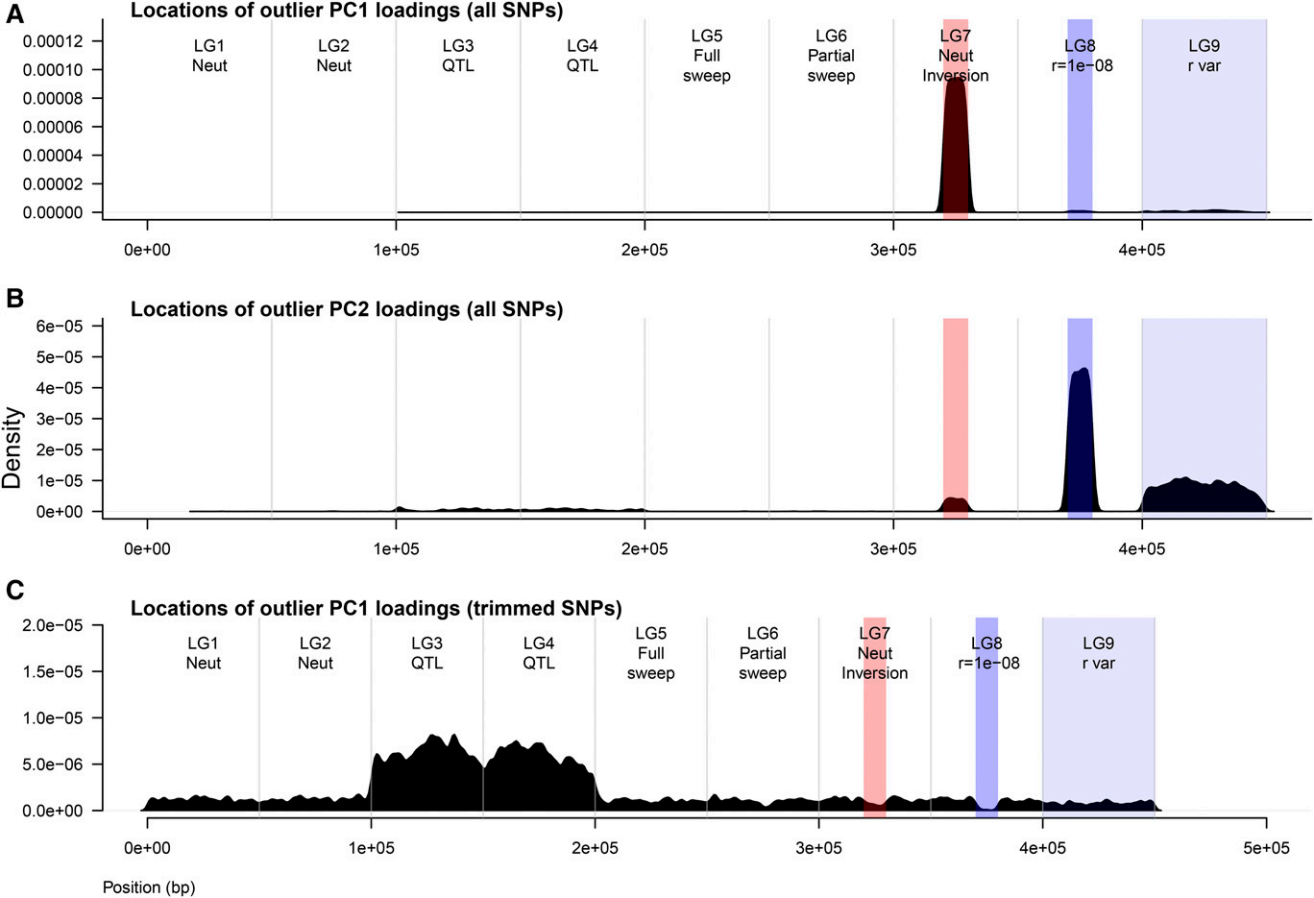
Loadings of loci onto PC axes across all replicate simulations. Each panel shows the frequency distribution of genomic locations that had outlier PC loadings for that scenario. A) When all SNPs were used, typically SNPs in the inversion would have outlier loadings along PC1 (corresponding to scores along x-axis in Figure 3A). B) When all SNPs were used, typically SNPs in one of the low recombination regions would have outlier loadings along PC2 (corresponding to scores along y-axis in Figure 3A). C) When a set of quasi-independent thinned SNPs were used, the QTNs that adapted to the environment (or neutral loci linked to them) had the highest loadings along PC1 (corresponding to scores along x-axis in Figure 3B).

The above results can be explained by understanding how loci load onto PC axes. When all SNPs in the data were used in the PCA to estimate population structure, in the majority of simulations the neutral inversion on LG7 loaded most strongly onto the first PC axis (Figure 4A). This explains why in Figure 3A, PC scores of individuals along PC1 could be explained by their inversion haplotype. The genomic location that loaded most strongly onto the second PC axis varied from simulation to simulation, but was typically the region of low recombination on LG8, or less frequently the region of recombination variation on LG9 or the neutral inversion (if it didn’t load onto the first PC axis) (Figure 4B). Thus, in Figure 3A, PC scores of individuals along PC2 were segregated according to their haplotype in the region that had the highest loading along this axis.

In contrast, when a set of thinned SNPs were used, the genomic regions that contained QTN that adapted to the environment (LG2 and LG3) had the highest loadings onto PC1 (Figure 4C). More specifically, the QTNs selected for local adaptation to the environment (or neutral loci linked to them - whichever were present in the trimmed set of SNPs) in each replicate simulation had the highest loadings. This explains why in Figure 3B, individual scores along PC1 reflected the isolation-by-environment structure along the environmental gradient.

In preliminary analyses with other methods based on using allele frequencies to assign individuals to ancestral populations (*e.g.*, STRUCTURE and similar methods, Pritchard *et al.* 2000; Frichot *et al.* 2014), I also found that only the estimates of population structure based on a set of thinned SNPs were accurate in describing the isolation-by-environment pattern present in the neutral data (results not shown).

### Performance of genome scans

#### Selective sweep methods:

**Figure 5:**
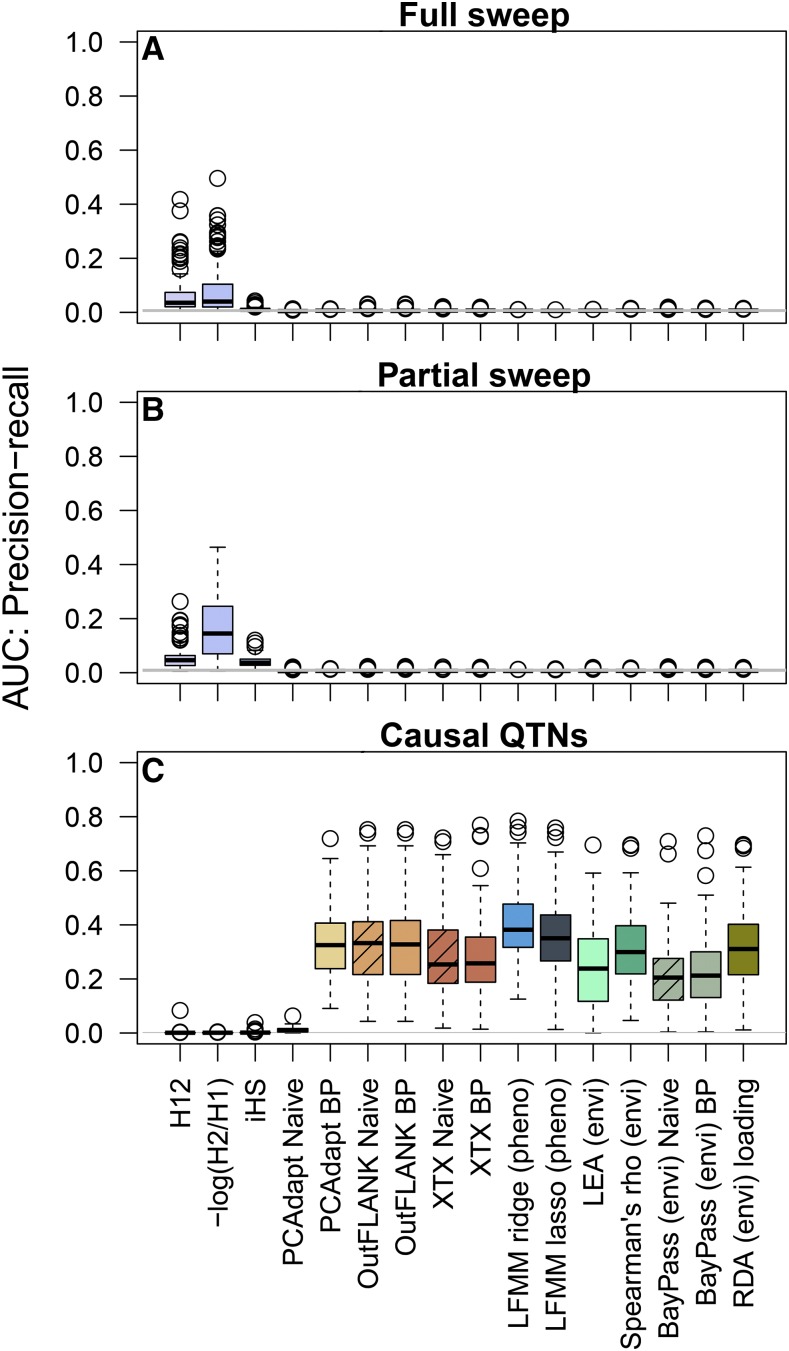
Area Under the Curve for Precision-Recall (AUC-PR) measures the overall performance of each statistic: AUC = 1 means that all causal loci have larger signals than neutral loci, and an AUC ∼0.01 is expected under random chance (horizontal gray line). A) AUC-PR evaluated in the region of the full sweep (2000 bp on either side). B) AUC-PR evaluated in the region of the partial sweep (2000 bp on either side). C) AUC-PR evaluated at all causal QTNs that explained greater than 1% in the additive genetic variance. Each method is shown in a different color, and the naive practice is shown with hatched bars when applicable.

**Figure 6:**
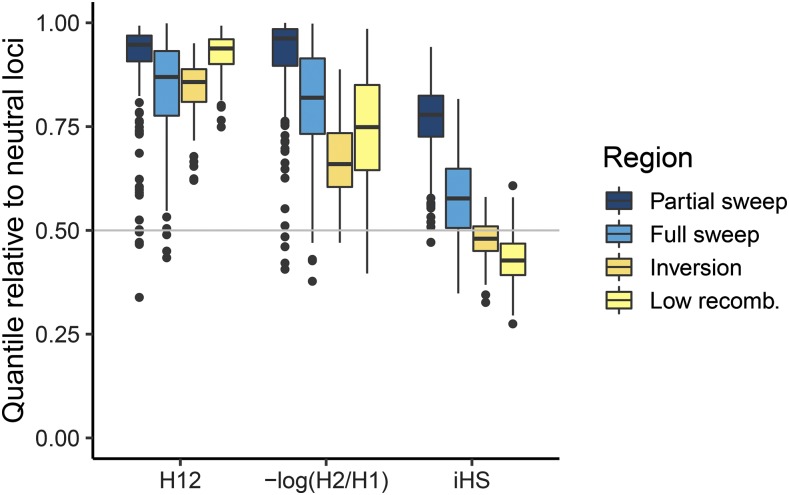
Boxplots of empirical quantiles of each test statistic in different regions of the genome. The genome-wide median at neutral loci (0.5 quantile) is shown as the horizontal line.

Performance was assessed as the Area Under the Precision-Recall Curves (AUC-PR). Sweep statistics generally had low performance (AUC-PR < 0.3) for detecting the full and partial sweeps (Figure 5 A and B, first three columns). The low performance based on AUC-PR could be because (1) low power to detect the sweep due to lack of signal around the sweep area, and/or (2) large false positive signals in other areas of the genome. These effects can be visualized by the empirical quantile of the signal in different regions of the genome relative to the neutral loci simulated on LG-1 and LG-2. For instance, *H_12_* and *H_2_/H_1_* had strong signals at the partial sweep (0.8-1.0 quantile) while iHS had a relatively weaker signal (0.6-0.9 quantile) (Figure 6). However, *H_12_* also had elevated signals in the region of the inversion and region of low recombination (>0.75 quantile), while *iHS* in these regions overlapped with the median signal at neutral loci (Figure 6, see also Manhattan plot in Supplementary Figure S5). Overall, *H_12_* and *H_2_/H_1_* had similarly large signals at the sweep mutations but also elevated signals in regions of low recombination rate, which led to lower overall performance. *H_2_/H_1_* outperformed *H_12_* in some cases because it was less sensitive to recombination variation (Figure 6) and neither statistic was affected by the minor haplotype frequency within the inversion region (Supplementary Figure S6). On the other hand, the low performance of *iHS* was caused by less power to detect the sweep mutations, even though it was not affected by recombination variation (Figure 6).

**Figure 7:**
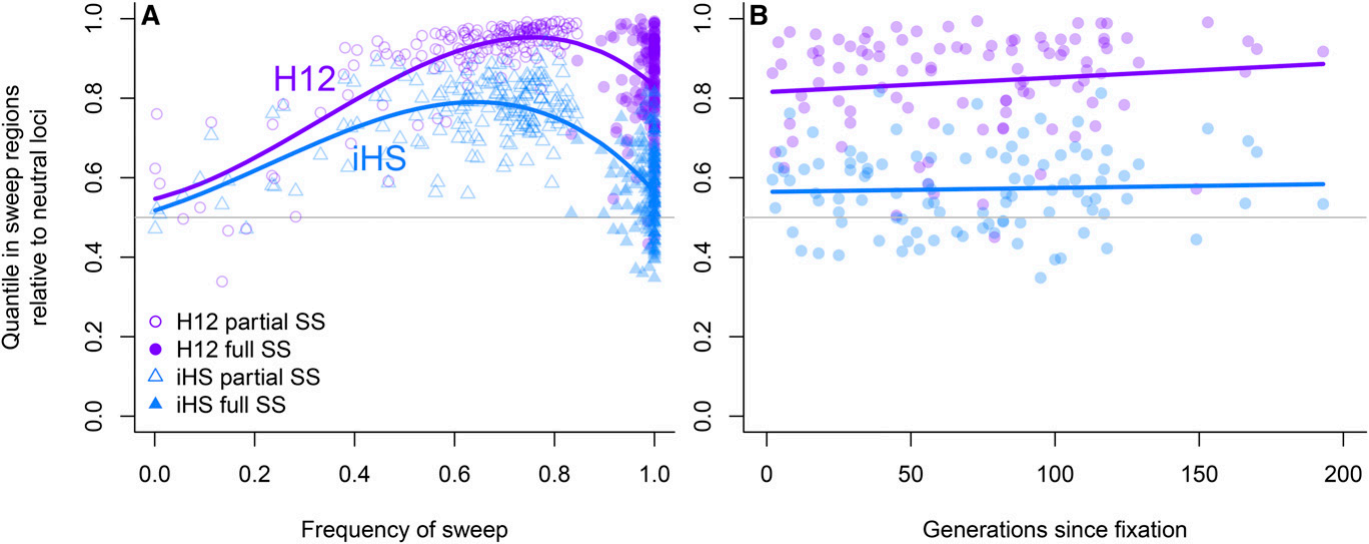
Empirical quantiles of *H_12_* and *iHS* across replicate simulations as a function of (A) allele frequency and (B) for fixed sweeps, the number of generations since fixation. The genome-wide median at neutral loci (0.5 quantile) is shown as the horizontal line.

The performance of all statistics depended on the allele frequency of the sweep mutation at sampling, with higher power of all statistics to detect sweeps that had not yet fixed. *H_12_* always had higher power than *iHS* across allele frequencies and after fixation (Figure 7). The power of *H_12_* was maximized at an allele frequency of 0.7-0.9, while the power of *iHS* was maximized at an allele frequency of 0.6-0.7 (Figure 7 A). Both statistics retained similar power between fixation and up to 200 generations after fixation (Figure 7 B).

#### Differentiation outlier methods:

**Figure 8:**
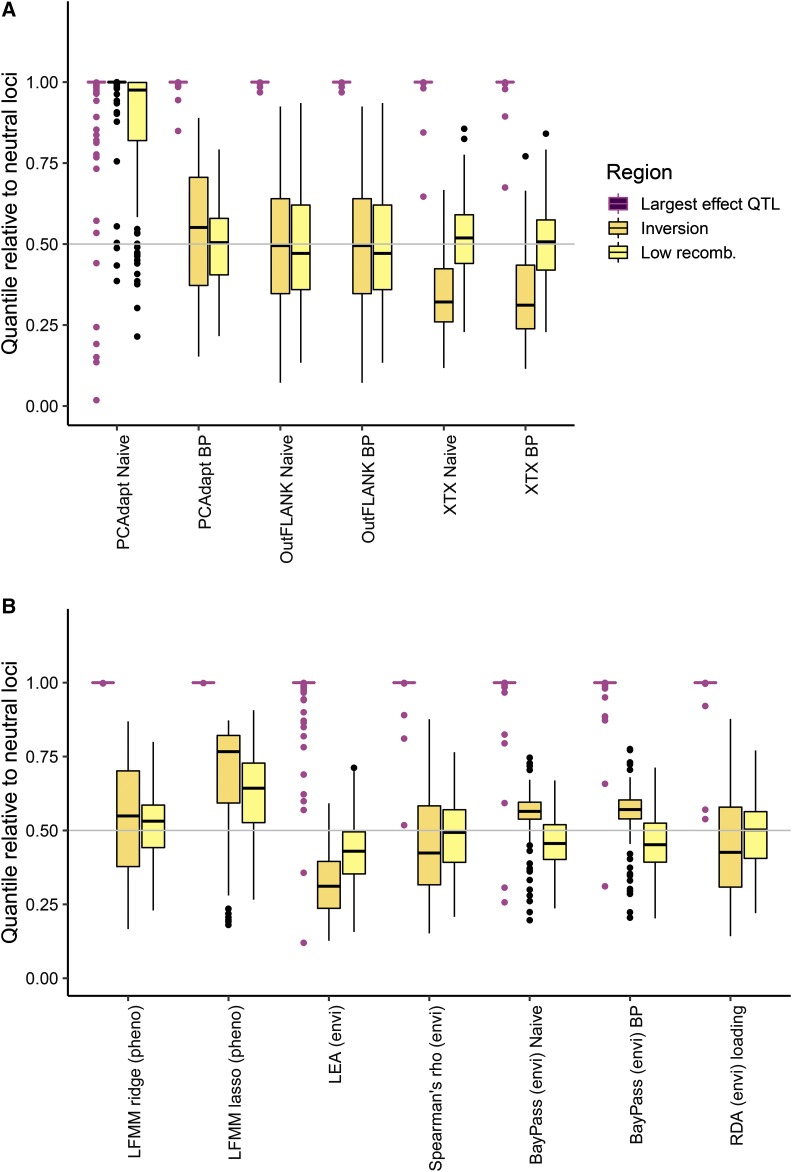
Boxplots of empirical quantiles of each test statistic in different regions of the genome. The genome-wide median at neutral loci (0.5 quantile) is shown as the horizontal line. (A) Differentiation outlier statistics, comparing the “naive” to the “best practice” (BP). (B) Association tests.

Both the naïve and best practice approach was evaluated for PCAdapt, OutFLANK (*F_ST_*), and BayPass (*X^T^X*). In general, OutFLANK and BayPass did not have a large difference in performance between the naïve approach and best practice (compare “naïve” to “best practice” for the two methods in Figure 5C and Figure 8A). However, the AUC-PR of PCAdapt increased greatly when the neutral population structure was computed on the set of thinned SNPs (best practice) rather than the entire set of SNPs (naïve approach) (Figure 5C for AUC-PR, compare PCAdapt “naïve” to PCAdapt “best practice”). This difference in performance occurred because using the naïve approach resulted in large false positive outlier signals in the inversion and regions of low recombination (Figure 8A, compare PCAdapt “naïve” to PCAdapt “best practice”). These outlier signals occurred in the naïve approach because of the way that regions of low recombination loaded onto the principal components (Figure 4).

The “best practice” for all methods had similarly moderate performance (Figure 5 C, AUC-PR ∼0.1-0.8), similarly large signals at the largest effect QTL in the data (Figure 8 A, quantile ∼1.0), and median quantiles in the inversion and low recombination regions similar to that expected based on neutrality (Figure 8A, median quantile ∼0.5 across replicate simulations). In some replicate simulations, a haplotype in a region of low recombination drifted to different frequencies among subpopulations and resulted in an elevated signal within that region (*e.g.*, Supplementary Figure S7, Manhattan plot). When comparing the methods for the “best practice” scenario using the AUC-PR, PCAdapt and OutFLANK (median AUC-PR ∼0.35) had slightly higher performance than BayPass (median AUC-PR ∼0.3, Figure 5B).

**Figure 9:**
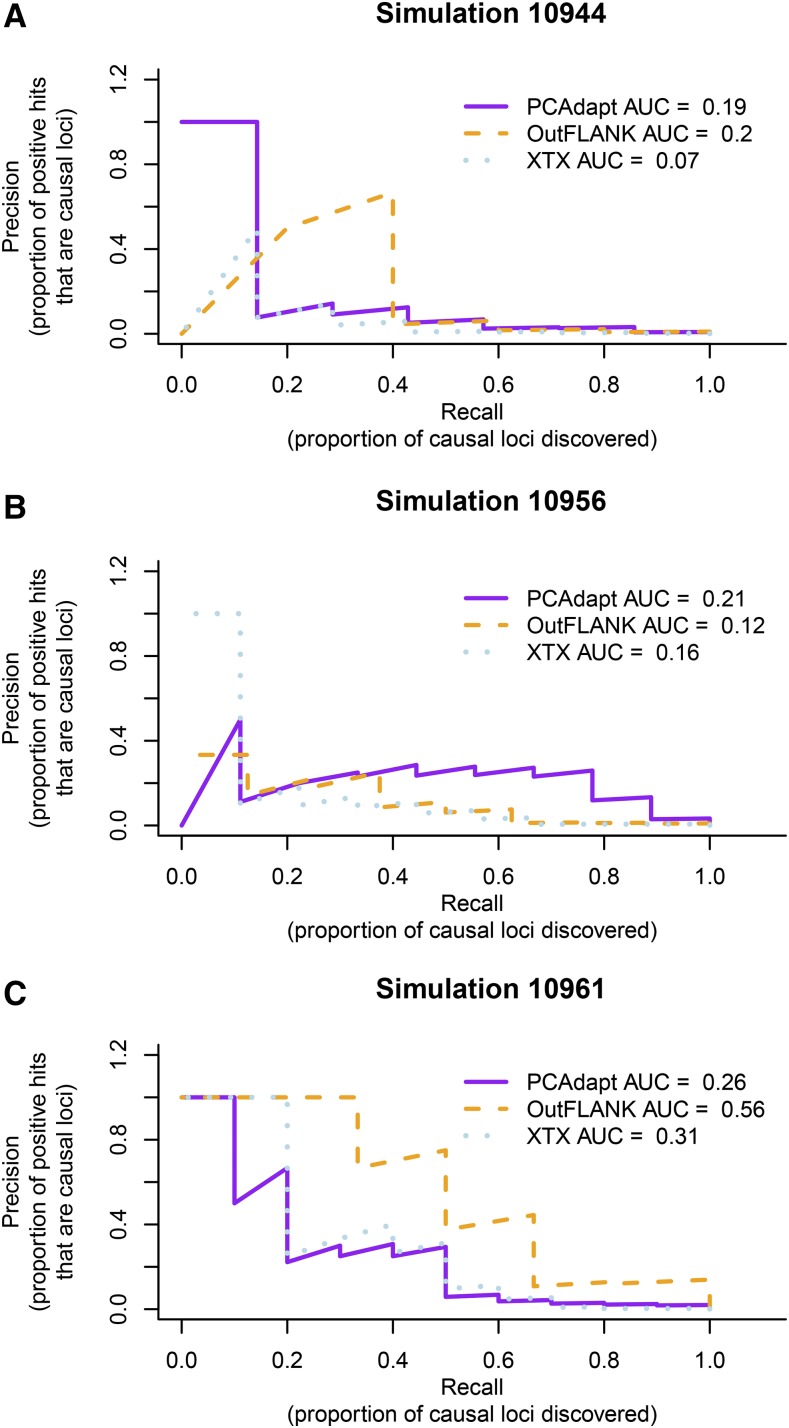
Precision-recall curves for three replicate simulations. For each method, the Area Under the Curve (AUC-PR) is indicated in the legend. Because large-effect loci tend to have the largest signal and be discovered first, the power of methods to detect them are captured whichever method has a larger area under the curve on the left side of the plot where recall is low (*e.g.*, only the loci with the largest signals have been discovered). The power of methods to detect moderate-to-small effect loci are captured by whichever method has a higher AUC-PR in the center and right side of the plot where recall is higher (more of the causal loci have been discovered). Precision decreases with recall because many neutral loci have signals that are larger than small-effect causal loci.

Overall, there was considerable variation from simulation to simulation in which differentiation outlier statistic performed the best and had the most power to detect smaller effect QTNs. For example, in Figure 9A OutFLANK had the best performance: although PCAdapt had higher precision for the largest-effect QTNs, OutFLANK had higher precision for moderate-to-small effect QTNs and this resulted in an overall higher AUC. However, in Figure 9B PCAdapt had the best performance: although *X^T^X* had higher precision for the largest-effect QTNs, PCAdapt had higher precision for moderate-to-small effect QTNs and this resulted in an overall higher AUC. In Figure 9C, OutFLANK had the highest precision to detect QTNs of all effect sizes. These results illustrate that no single statistic is ideal in all scenarios.

#### Association methods:

For associations with phenotypes (GWAS), ridge regression outperformed lasso regression for latent factor mixed models (LFMM, Figure 5C). The lower performance for LFMM-lasso occurred because although both methods were able to detect the largest effect QTN (Figure 8B), the lasso sometimes had elevated signals in the inversion and the regions of low recombination (Figure 8B, see also Manhattan plot in Supplementary Figure 8).

For associations with environments (GEA), Spearman’s *ρ* and RDA had similarly high performance, followed by LEA, and then BayPass (*BF*) (Figure 5C). All methods had extreme signals at the largest effect QTN and were not affected by recombination variation (Figure 8B). Although BayPass (*BF*) showed a slightly elevated signal in the inversion compared to the genome-wide median (Figure 8B), these signals were far below anything that would be considered significant (average log_10_(*BF*) in the inversion was -11.2; for a log_10_(*BF*) to be considered “decisive evidence” it should be larger than 2 (Kass and Raftery 1995)). Finally, it should be noted that in some replicate simulations a haplotype in a region of low recombination could drift to different frequencies among populations and show elevated signals for some association tests but not others (Manhattan plot in Supplementary Figure S8).

Finally, the SNP loadings on the unconstrained axes of the RDA (*e.g.*, the principal components) reflected genomic variation in recombination rate, with the inversion typically loading onto the first PC and the other regions of low recombination loading onto the subsequent PCs (Supplementary Figure S9).

## Discussion

Genome scan methods have different strengths and weaknesses, and it is important to understand what these are when detecting signals of selection in genomes. This study used the process of analysis validation (Lotterhos *et al.* 2018) to illustrate how inversions and regions of low recombination can confound some genome scans. Methods designed to detect selective sweeps based on haplotype frequency may show elevated signals in regions of low recombination. Non-independence among SNPs due to LD can disproportionally affect estimations of population structure, especially those based on principal components. For some differentiation outlier and association methods, even a small percent of non-independent SNPs in the data can bias estimates population structure and create false positive signals in these regions. This negative consequence can be mitigated if a set of SNPs that have been thinned for LD are used to first estimate population structure or calibrate the null distribution, which better captures the neutral demography of the study species. Given the importance of inversions (Schwander *et al.* 2014; Thompson and Jiggins 2014) and centromeric regions (Berner and Roesti 2017) in local adaptation, these results illustrate the importance of validating genomic pipelines on simulations before making conclusions about the genomic architecture of adaptive traits.

### Estimating population structure with principal components

Many seminal studies that have been used to evaluate genome scans have used independently simulated SNPs to evaluate the methods. In the seminal paper by Price *et al.* (2006) where principal components were first introduced as a way to control for population structure in GWAS, they simulated an independent set of SNPs to evaluate the method. Following this seminal paper, a few studies have illustrated some of the caveats of using principal components to estimate genetic structure using independently simulated SNPs. For instance, Novembre and Stephens (2008) showed that PCA can depend on the details of a particular dataset, including distribution of sampling locations and amounts of data. Similarly, McVean (2009) showed that PCA can be strongly influenced by uneven sampling among populations and biases in SNP ascertainment, and that the accuracy of PC axes to capture structure increases with the number of independent SNPs.

By illustrating the effects of genomic heterogeneity in recombination on principal component estimates of genetic structure, this study builds on these previous studies as well as ground-truths empirical studies that have documented the effects of linkage disequilibrium on PCA. For instance, the Wellcome Trust Case Control Consortium (2007) found that data from a 500K human SNP chip was dense enough to create high loadings of SNPs onto regions of linkage disequilibrium, and as a result four of the first six PCs on population structure reflected local genomic LD. They also showed how related individuals can distort projections of samples onto PC axes and admixture proportions. Similarly, Price *et al.* (2008) articulated concerns about how long-range LD and inversion polymorphisms can produce signals of unusual ancestry and should be accounted for in genome scans for selection. Privé *et al.* (2018) showed how long-range LD regions from a celiac disease dataset (∼300K SNPs) load onto principal components and how these effects can be removed with SNP thinning. Most recently, Li and Ralph (2019) showed that inversions and regions of low recombination can dominate patterns of mean relatedness along the genome as determined by local principal components.

Three main conclusions can be drawn from the simulations presented in this study and empirical studies. First, for each PC axis, individual scores will be dominated by the SNPs that have the highest loadings along that axis. Second, using a SNP dataset that has *not* been trimmed for LD may result in local genomic LD loading onto some of the principal components. On one hand, this may be a good way to identify local regions of LD, and to quickly visualize and group individuals according to their haplotype within that LD region (for an empirical example see Barney *et al.* 2017). Even low density SNP datasets (consisting of tens of thousands of SNPs) may be susceptible depending on the range of LD; for example the resolution in these simulations was a SNP only every 0.07 cM (∼50 cM per LG divided by ∼700 SNPs per LG), which corresponds to every ∼70,000 bp in humans (corresponding to a 50K SNP chip). Third, thinning SNPs for LD can more accurately capture neutral genetic structure (although scores will still be dominated by SNPs with the highest loadings). These caveats and best practices will be useful to those using various kinds of software that incorporate principal components (PCs) of genotypes (Abraham and Inouye 2014; Abraham *et al.* 2016; Galinsky *et al.* 2016; Price *et al.* 2006).

### Variation in performance of genome scans

This study compared the ability of three statistics to detect hard selective sweeps from new mutation. In these simulations, *iHS* statistic overall had low power than *H_12_* and *H_2_/H_1_* to detect the sweep across allele frequencies. This contrasted with previous studies that found *iHS* had high power to detect partial sweeps that had not reached fixation (Tang *et al.* 2007; Schrider *et al.* 2015; Schlamp *et al.* 2016). *H_12_* and *H_2_/H_1_* had similarly large signals at the hard sweep across a range of allele frequencies, which is in agreement with previous studies that found these statistics had high power to detect the a mutation under strong selection simulated here (Schrider and Kern 2016). However, both statistics also showed elevated signals in the inversion and regions of low recombination and this led to overall lower performance. In the simulations these neutral regions were dominated by only a couple haplotypes segregating in the population, and so statistics based on combining the frequencies of the two most common haplotypes were inflated. In *Drosophila melanogaster H_12_* peaks were not associated with inversions (Garud *et al.* 2015), which suggests that the results from this study may not apply to all cases.

Of the three differentiation outlier methods tested with these simulations, the naïve PCAdapt was shown to be the most susceptible to genomic variation in LD, but when best practices were employed this method had similar performance to other methods. This sensitivity for the naïve approach occurred when all SNPs were used to estimate the principal components, because regions of high LD had high loadings on the PC axes. OutFLANK and BayPass were not sensitive to the set of SNPs used. However for OutFLANK, it should be noted that bias in the estimation of the neutral mean *F_ST_* and *df* has been observed with high-density genomic data compared to a subset of trimmed SNPs (KEL, pers. obs.), and that best practices should still be employed. Previously, the model that BayPass is based on was shown to perform poorly under isolation by distance compared to other methods (Lotterhos and Whitlock 2015), and this should be considered when interpreting the results presented here.

Almost all the association methods had similar performance. There was some variation in the behavior of three latent factor mixed models (the ridge regression with phenotype, lasso regression with phenotype, and LEA with environment), with the lasso regression sometimes showing outlier signals in the inversion or regions of low recombination. In the implementation of these models in the R package, the ridge regression is solved analytically while the lasso regression is solved numerically, which may indicate that the ridge estimates are more accurate (Caye *et al.* 2019). However, in some of the evaluations performed by Caye *et al.* (2019), the lasso regression appeared to outperform the ridge regression (*e.g.*, in the Celiac disease dataset), and so additional validation may be required for these implementations to understand their different strengths and weaknesses. Although both Spearman’s *ρ* and redundancy analysis had high performance to separate signals of selection at QTNs from signals at neutral loci, it is important to note that these methods do not correct for population structure: even though causal loci have large signals than neutral loci, many neutral loci can have significant *P*-values when structure is not accounted for (Meirmans 2012).

### Limitations of simulations

While the simulations presented have certain types of realism in terms of including local adaptation, selective sweeps, and recombination variation, each type of realism was simulated on discrete linkage groups. Such compartmentalization of genomic heterogeneity is unlikely to be found in nature. Additionally, natural inversions may show evidence of exchange between arrangements, which may occur during rare double crossovers or gene conversion events (*e.g.*, Schaeffer and Anderson 2005). This type of exchange was not captured by the simulations. The simulated data were also idealistic in that every individual in the population was sampled, their genotypes were known without error, and there was no missing data. There was also no environmental noise that affected the phenotypes (which were also known exactly without error), which is part of the reason for the high performance of GWAS methods. While these properties of the data are idealistic, the effects of sampling and genotyping errors could easily be explored with the archived files. Since these simulations used scaling of mutation and recombination rate to population size, they may fail to capture some important dynamics that are unique to large samples. The ability of genome scans to produce accurate results can also be affected by a number of qualities of the data, including sampling strategy (Lotterhos and Whitlock 2015), ascertainment bias (Lachance and Tishkoff 2013), allele dropout (Gautier *et al.* 2013), and missing data.

### Selection in regions of low recombination

Many empirical studies suggest that inversions may capture sets of adaptive QTNs, deeming these regions “supergenes” (Cheng *et al.* 2011; Schwander *et al.* 2014; Thompson and Jiggins 2014; Kapun *et al.* 2016; Barney *et al.* 2017; Berg *et al.* 2017). Others have similarly inferred that centromeric regions of low crossover rate facilitate adaptive divergence (Berner and Roesti 2017). Do the simulations results suggest that the importance of “supergenes” or regions of low recombination have been overstated in the literature? Robustly answering this question would require a thorough meta-analysis of how these regions were identified, which methods were used, and whether the authors employed best practices when scanning the genome. Many of the classic examples of inversions underlying adaptive differences among populations or phenotypes within a species are from traditional QTL mapping, which was not evaluated in this study. However, the association tests in this study tended to perform well and not be susceptible to false positive signals from the inversion or regions of low recombination. In general, caution should be applied in drawing conclusions about the importance of a specific genomic regions based solely on the functions and ontologies of genes located within that region (Pavlidis *et al.* 2012) or based solely on chromosomal structure.

When applicable, employing the “best practice” suggested here should help discern false positive signals in neutral inversions from true positive signals in adaptive inversions. Models show that locally adapted inversions have higher genetic differentiation than the genome-wide background or drifting inversions (Guerrero *et al.* 2012, but note neutral inversions under genetic drift can produce coalescent patterns similar to locally adapted inversions of intermediate age). The ability of methods to discern different genetic architectures (*e.g.*, monogenic *vs.* polygenic) within adaptive inversions, however, is an important area for future research, and one that could give important insights to the “supergene” hypothesis. Discerning whether an adaptive inversion has a monogenic or polygenic basis may be a difficult task with population genomic data because of the extended signals resulting from low recombination rates within the inversions. However, new approaches based on machine learning are being used to discern among different types of genomic heterogeneity, including different types of selection acting on a single sequence, and may hold promise for this problem (Schrider and Kern 2018).

### Conclusions and best practices

The single best practice that can be employed when analyzing genome scans for selection is to validate your genomics pipeline (including filtering, imputation of missing data, genomic realism, and analysis methods) on simulated data (Lotterhos *et al.* 2018). In most cases this will not feasible, as it could be as much work as the empirical study itself. The following steps may help to validate analyses with genome scans when simulations are not available. First, related individuals should be removed from the analysis (*e.g.*, Wellcome Trust Case Control Consortium 2007) or the relatedness should be accurately controlled for in the statistical test. Next and when applicable, estimate the neutral population structure (either by principal components, a covariance matrix, or by estimating parameters on some model) on a quasi-independent set of SNPs that has been thinned for LD. When applicable, use this same subset of SNPs across all methods to control for population structure in testing for selection across all sites in the data. When applicable, also inspect *P*-value histograms and *Q-Q* plots to check assumptions, and calculate the genomic inflation factor (François *et al.* 2016). Next, evaluate the sensitivity of your results to the decisions made in the pipeline, such as filtering SNPs, imputing genotypes, phasing haplotypes, etc. The effects of unequal sample size should also be evaluated to ensure that they do not affect the results (McVean 2009).

Finally, datasets and pipelines that reproduce the results should be required for publication, including widely used file formats (*e.g.*, VCF) that are produced after the process of filtering reads, mapping reads, and calling variants. Only when easy-to-reproduce pipelines are provided will it be possible to re-analyze data if a weakness is identified in one of the methods used in the pipeline.

## Appendix

### Fitness component: competition.

Individuals that experienced more competition had a lower fitness component for competition. Competition for a single individual was experienced as the sum of interactions with all other individuals, which declined with distance and increased with phenotypic similarity. Total interaction strength (*P_Ci_*) for any individual *i* at spatial location *x_i_*, *y_i_* equaled*:*

$$P_{Ci}=\sum_{j=1,i\neq j}^{N}\frac{\exp\left(-\frac{1}{2}{\left({\text{X}}_{j}-{\text{X}}_{i}\right)}^{T}\Sigma^{-1}\left({\text{X}}_{j}-{\text{X}}_{i}\right)\right)}{\sqrt{2\pi \;\Sigma }}$$ (2)

Where ${\text{X}}_{i}$ = [ *x_i_*, *y_i_*, $\rho_{i}$] of individual *i*, $\rho_{i}$is the phenotype of individual *i*, ${\text{X}}_{j}$ = [ *x_j_*, *y_j_*, $\rho_{j}$] for individual j≠i, and Σ was a 3 x 3 diagonal matrix with equal variance $\sigma_{C}$ and no covariances. Thus, $\sigma_{C}$ described the spatial and phenotypic distance over which individuals interacted. The competition fitness-component for individual *i* was estimated from *P_Ci_* as an exponential decay ($\omega_{Ci}$ = exp(0.0009*100**P_C_**(1-*P_C_*))), such that individuals that had 0 total interaction strength had a relative fitness of 1 and individuals that had large interaction strengths (> ∼7) had a total fitness of about 0. In this manner, local density dependence was implemented in the model and the maintenance of some genetic variation was maintained within genetic neighborhoods (*e.g.*, weak disruptive selection).

### Fitness component: mate choice.

Individuals also experienced mate choice. The total interaction strength for mating for individual *i* (*P_Mi_*) was also given by Equation 2, such that individuals that were closer in space and phenotype had larger interaction strengths, with the exception that Σ was modeled with a different variance ($\sigma_{M}$). The mating fitness-component ($\omega_{Mi}$) was equal to *P_Mi_* scaled to relative fitness for this component. Thus, assortative mating favored the erosion of genetic variation (*e.g.*, weak stabilizing selection) within local neighborhoods.
